# Atlanta Academy of Medicine

**Published:** 1875-03

**Authors:** 


### Atlanta Academy of Medicine.

ROBERT BATTEY, M.D., REPORTER.

ATLANTA, GA., 26th January, 1875.

Dr. John M. Johnson, president, in the chair.

Dr. Logan called attention to a recent case of diphtheria in a child four years old, attended by a fading eruption and greatly depressed pulse, with other evidences of nervous prostration, brought from Jefferson county, Georgia. Treated by local stimulation, with the usual internal remedies, with success. He directs attention to the conjunction of the diphtheritic membrane with an eruption rather scarlatinal in character. The young lady who had charge of the child was subsequently attacked by the same malady in milder degree, but unaccompanied by eruption. Two boys in the family subsequently developed the same disease, with eruption resembling roseola upon the body and extremities, not the face. Is in doubt as to the precise diagnosis of the disease. The disease of the mucous surfaces was evidently diphtheritic. Query: Is this diphtheria with merely an incidental eruption, or is it scarlatina with diphtheritic complications? The treatment was chiefly turpentine, chlorate potassium and creosote. The incubation in the cases of the boys was about ten or twelve days. Those which had the eruption most showed excessive nervous prostration. The first case came from Jefferson county, and was seen by Dr. E. H. Hunter, of Louisville.

Dr. W. F. Westmoreland thinks the cases diphtheria. A careful examination of the deposit would be necessary to establish the diagnosis. This can readily be done by inoculation upon one of the lower animals, as a rabbit, for instance. Suggests that this test be instituted, and also the examination of the deposit under the microscope.

Dr. Logan is not prepared to admit that vegetable spores are pathognomonic of diphtheria. It is not settled that the disease is parasitic. Thinks the malady a blood disease and not parasitic.

Dr. Westmoreland thinks that eminent German pathologists are settled in the opinion of the parasitic character of this disease. Thinks the membrane of croup is an exudation, while the the false membrane of diphtheria is a deposit. Some authorities regard the constitutional symptoms primary; others contend that the local symptoms are primary. Thinks the failure to find bacteria may arise from imperfection in the microscopy or mistake as to the diphtheritic character of the false membrane.

Dr. Logan desired only to be understood that, in his opinion, this question is not yet definitely settled.

Dr. Westmoreland thinks we are making rapid progress in the solution of the question.

In response to a question of the president, Dr. Logan stated that the eruption was a slightly elevated rash.

Dr. Taliaferro inclines to the diagnosis of scarlatina. The eruption appears to have been a distinctive feature of the epidemic in Jefferson. The deposit upon the throat may not have been diphtheritic at all. The Germans of late have had a "fungus mania"—attempting to explain everything upon the fungus theory. Does not regard diphtheria as contagious—only epidemic.

Dr. J. T. Johnson thinks the German pathologists have run wild of late upon their fungus theories. They differ so widely among themselves as to greatly weaken professional confidence.

Dr. Westmoreland objected to Dr. Johnson's term "German theories;" he should call them German *facts*.

Dr. Taliaferro once had much confidence in the German fungus theory of malaria, but found to his sorrow that it would not bear the test of experience. He abandoned quinine for the sulphites, but soon found it necessary to retrace his steps, or lose public confidence.

Dr. Westmoreland in response to a question expressed his belief that we will always find bacteria where we find diphtheria. Thinks the Germans are on the right track and in the direction of progress. Thinks the microscope of the greatest value in the progress of these investigations, and likely in the future to develop the grandest results in the cure of disease.

Dr. Woodhull remarked upon the close interchangeability between scarlatina and diphtheria. He regards the two as merely different manifestations of the same disease. Asked if sulphurous acid had been tried in diphtheria. If the disease is cryptogamic it is indicated.

Dr. J. T. Johnson replied that it had been for six years, and with no very marked results.

Dr. Armstrong thought the cases not diphtheria, but a contagious disease. Does not regard diphtheria as contagious. He thinks the cases were scarlatina. It is not very uncommon to find exudations in the throat in scarlatina.

Dr. Hall, of Warren county, being called upon, expressed the opinion that the cases described by Dr. Logan are diphtheria. Has seen the eruption not unfrequently in diphtheria. Regards the eruption as an expression of the intensity of the disease.

Dr. J. G. Westmoreland saw the cases. Does not think them cases of scarlatina. Does not think scarlatina so very contagious as is generally supposed. Is not clear in his mind upon the question of diagnosis.

Dr. Griggs, of West Point, remarked that the strawberry appearance of the tongue in scarlatina he regarded as characteristic; the eruption also is peculiar and characteristic, consisting of a diffuse erythematous redness with the elevated points interspersed. The eruption in diphtheria is erythematous merely, and usually appears towards the close of the disease. Regards both diphtheria and scarlatina infectious. The infection depends upon the intensity of the disease on the one hand, and the power of resistance on the other. He accepts the cryptogamic theory of the Germans with reference to diphtheria. Thinks constitutional treatment, of supporting character, of first importance in diphtheria. Local applications should not be energetic, or they do harm rather than good. Thinks the cases under consideration were diphtheria, complicated with roseolous eruption.

The president, upon call, remarked that we sometimes find scarlatina and diphtheria intercurrent; they are cognate diseases. His own son had a severe and decided attack of scarlatina anginosa in December last, with exudation upon the tonsils diphtheritic in character. The disease did not spread at all in the house. The nurse had a light attack of scarlatina, but it did not spread to other children in the house in contact with the patient. He does not regard either scarlatina or diphtheria eminently contagious, as many esteem them. Thinks Dr. Logan's cases are diphtheria, and the eruption rather an accidental complication. Treats diphtheria with stimulants, nourishing food, tinct. iron in large doses, and quinine; gargles of chlorate potassa and glycerine to keep the throat moist, and solutions of sulphate of copper as an application to the throat. Attaches much importance to keeping the throat moist with the chlorate potassium and glycerine. Does not believe in local caustics; employs twenty grains sulph. copper to ounce of water as a stimulant to the throat.

Dr. Logan, upon call, expressed much doubt as to the diagnosis; he, however, is inclined to regard them as scarlatina.

Dr. Todd regards the cases as diphtheria.

Dr. Rudicil, of Chattooga, has treated similar cases at home. He uses stimulants, quinia and iron, with usual local applications. He regards the cases reported as diphtheria. Attaches importance to stimulants.

Dr. Baird testified from his experience in his own person to the great relief afforded by gargles of chlorate of potassium. Has no confidence in the curative effects of local applications in diphtheria; they are only useful as palliatives.

Dr. W. F. Westmoreland remarked upon the great discrepancy of opinion expressed upon the subject of contagion. He has no confidence in tracheotomy in diphtheria. Thinks we should seek to destroy the fungi; we ought to favor inflammation in diphtheria rather than seek to subdue it; produce effusive inflammation to guard against absorption; give alcoholic stimulants, quinine and iron, to keep up the powers of life. Antagonize absorption of the parasites and support the powers of life, is the treatment in a nutshell.

Dr. Battey, on call, remarked upon the rarity with which we find disease conforming to the precise types as laid down in the books. Thinks it much more important to direct our minds to antagonizing the tendency to death, rather than to the learned classification of disease. He is not fully satisfied himself with the diagnosis of either diphtheria or scarlatina. He is struck with the resemblance of Dr. Logan's cases to the epidemic prevailing some months ago in Cobb county and North-western Georgia. Adjourned.

---

JAMES B. BAIRD, M.D., REPORTER.

---

ATLANTA, GA., February 9, 1875.

V. H. Taliaferro, M.D., Vice-President, presiding.

Dr. J. G. Westmoreland, the essayist of the evening, read a paper on the physiological action and therapeutical results of mercury; in which he set forth the desirability of classifying remedies according to their physiological effects, and objected to the results of experiments made, with reference to the effect of mercury, on healthy dogs. Mercury may diminish the flow of bile from a healthy liver, but holds that it increases it when the liver is congested and inactive.

Dr. Logan objected to the term physiological as applied to the action of a remedy on a diseased organ. Any relation between a remedial agent and a diseased organ is not physiological.

Dr. J. G. Westmoreland explained that the physiological action of a remedy is the effect it exerts on an organ, regardless of disease. He cited, as an illustration, the effect on the stomach of an emetic. He objected to a classification based on the action of remedies. For instance, some are called diuretics; none are invariably so. Oil of turpentine, in certain debilitated conditions of the kidneys, excites them to increased action. When they are congested, on the other hand, it diminishes the amount of urine. Under his classification it would be called a renal stimulant.

Dr. Woodhull thought the point made by Dr. Logan well taken. Physiological action is confined to the processes of nature. When these actions are influenced by a foreign agent they cease to be physiological.

Dr. Taliaferro regarded the term used by Dr. Westmoreland as peculiarly fortunate. The normal effect of the remedy is expressed by it. Its natural or peculiar property is its normal or physiological action.

Dr. Logan, for this very reason, regarded the term peculiarly inappropriate. Physiology treats of the laws of vitality, either animal or vegetable, and cannot properly be applied to dead matter.

Dr. J. G. Westmoreland replied, on that idea the term was appropriate, for remedies modify vital actions. They either lessen or increase them.

Dr. Battey called attention to a specimen on the table, stating that it was an ovarian tumor removed by him that day. He desired to present the specimen while fresh, and therefore would proceed to report the case.

Mrs. ——, æt. 43, from Gwinnett county, mother of several children. About three years ago she discovered in the left iliac fossa a lump about the size of a hen's egg. It was firm, continued to grow steadily, though it caused no serious inconvenience under a year; then she experienced pain from it. It changed its position to the hypogastric region, becoming central, and after that causing a good deal of pain, continuing to do so until the present time. On the tenth of last June was called to see her. Found her bed-ridden, emaciated, and appeared worn out. After seeing her, was so thoroughly impressed with the idea that she could not survive for a month, that he considered it cruel and unnecessary to subject her to a critical examination. About a month later her case seemed to reach a crisis; she went on down; despaired of ever getting up. About the middle of July dysenteric symptoms were observed. These were tenesmus and bloody mucous discharges. In forty-eight hours a copious discharge from the bowels occurred. Was unable to describe the character of this discharge. Her friends identify it as similar to that evacuated from the cyst to-day. This continued for about a week. After he saw the case the abdomen increased rapidly in size. It was considerably reduced after the week of this discharge, and when relieved of distension she was greatly comforted, and the pain and general disturbance were less. She improved so much that she not only left the bed, but visited friends in Forsyth county. About a month ago he learned that she had gained flesh and strength, so he sent for her that he might make the diagnosis. Three weeks ago she arrived in the city. He, aided by Dr. Logan, examined the tumor, which was found to be in the second stage, according to Spencer Wells, rising above the pelvis, the summit reaching to the umbilicus. It projected forward, was easily movable, the abdominal walls slipped readily over its surface. The tumor was not tied down, but was freely movable upward and downward, indicating comparative absence of pelvic adhesions, and a long pedicle. Two fingers were passed between the uterus and tumor, proving that no intimate adhesions existed. The uterus was retroverted by pressure from the tumor. The examination was repeated several days later in company with Dr. W. F. Westmoreland, but the presence of the menses and some soreness from the previous examination prevented the same thoroughness that was previously observed. It was evident that there was a cyst of considerable size at the upper portion on the left side, and a cyst of less size at the upper portion on the right side. Fluctuation was less toward the base, showing increased thickness of the walls. The evidence was indisputable that the tumor was multilocular, that there was some solid matter also, and that adhesions to the large intestine, possibly the rectum, or not high above it, existed. Based this opinion upon the occurrence of dysentery and the discharge. If the small intestine had been involved thinks she would have had diarrhœa instead.

It was not the most favorable variety of tumor for an operation: first, it was multilocular; secondly, large amount of solid matter; thirdly, adhesions to the large intestine. He determined, however, as the general condition was favorable, no parietal or firm adhesions and a long pedicle, that an exploratory incision would be justifiable. Last Tuesday was appointed for the operation, but her menses being prolonged, and at the same time the presence of severe pleurodynia, and the prevalence of erysipelas in the city, caused him to defer it. Put her on quinine and iron. To-day her morale was good, and her general condition favorable, so the operation was performed, Drs. Logan, Westmoreland, Baird, Kendrick and Griggs, of Atlanta, Holmes, of Rome, Clements, of Chattooga county, O'Daniel, of Twiggs county, and Little, of Gwinnett county, present and assisting.

The patient received a stiff dram of whisky a half-hour before the operation. She was placed on the table and etherized. She came very soon under the influence of the anæsthetic—unusually so. The abdomen was opened by a short incision, three and a half or four inches in length, down to the peritoneum. The tumor, after the division of the peritoneum, was brought into view, and presented a dense fibrous wall. A long silver sound was swept over the surface and around the margins of the tumor, and showed the absence of adhesions as far as could be reached. The fingers were introduced into the right iliac fossa; no adhesions; then into the left iliac fossa, encountering the pedicle, long and narrow. The fallopian tube was attached to the pedicle and also a zig-zag convulated mass that to the touch seemed to be distended veins, but upon tracing it up was satisfied that it was a duplicature of the sigmoid flexure of the colon firmly adherent to the pedicle of the tumor. About this time Drs. Kendrick and Baird, who had charge of the anæsthetic, announced that she had ceased to breathe. She was immediately turned across the table, her head depressed and body elevated. Her pulse was reported good, notwithstanding the cessation of the respiration. The dependent position was maintained and breathing soon returned. Was unable to say how long the cessation of the respiration continued, though it was evident from the strength of the pulse that her condition was not one of extreme collapse. So intimate was the adhesion between the intestine and tumor that it was deemed best to encroach on the base of the tumor and cut out a portion and allow it to remain attached to the pedicle. This portion was transfixed by needle and double ligature, and tied so as not to implicate the bowel, thus forcing it to slough off. The pedicle felt as if it were twisted upon itself, like a cotton rope. This proved to be the case when it was brought into view. It was rotated upon its own axis one and a half or two times. This twisting of the pedicle is not uncommon, and sometimes is the source of fatal inflammation. The rotation in this case has not occurred recently—that is, within the last three weeks; for the position of the tumor was identical to-day with that occupied when first examined. A somewhat extensive and intimate adhesion existed between the upper part of the tumor and the omentum. It was deemed advisable not to attempt a separation on account of its firmness, so the omental attachment was ligated *en masse,* and separated with scissors. After emptying various cysts the tumor was readily drawn out through the incision, that had been extended to five inches. The pedicle was brought out at the lower part of the incision. The sigmoid flexure, after healing, will adhere to the cicatrix. The omental stump was brought out at the upper portion of the incision. The edges of the wound were brought together by ten points of silver suture, a tent was placed in the lower angle of the wound. This was put in on account of the necessary haste in concluding the operation. He was not able to cleanse the cavity as thoroughly as he desired, though care was observed throughout the operation to avoid the introduction of fluid into the abdomen, by packing sponge around the orifice, and he thinks but little fluid made its way into the cavity. If, however, it should be necessary, Douglas's fossa can be syringed out after removing the tent, and any septic material be brought away. The stump was dressed with water dressing and the patient put to bed. A little brandy, water and laudanum was given, but soon vomited. One-fourth grain of morphine was administered hypodermically. During the afternoon she has been comfortable. At three o'clock pulse 76, increasing in volume; at six o'clock, 85. Reacted well.

The tumor consisted of probably six cysts of notable size and some smaller. The first was punctured through its outer wall, and the others through the septa. Probable weight of tumor twelve pounds. The contents of the different cysts varied. In one the fluid was of a dark chocolate color and consistency. In the one on the right side it was clear yellow. Detritus of a blood-clot in the largest cyst proved the occurrence of an old hemorrhage.

In answer to an inquiry from Dr. Battey, Dr. Baird stated that he thought the period of the stoppage of respiration did not exceed thirty seconds.

Dr. W. F. Westmoreland read an essay on electricity and its therapeutical effects.

Dr. Logan inquired if there were any well established facts respecting the real efficacy of electricity in the treatment of disease. Expressed a want of confidence in its value.

Dr. Westmoreland replied that it was yet in its infancy and we know little about it.

Dr. Woodhull inquired as to its specific influence in pathological conditions, and especially its effect in spinal irritation.

Dr. John G. Westmoreland regarded it as a substitute for the nervous fluid, to which it acts somewhat similarly. In spinal irritation there is a deficiency of nervous fluid, and electricity supplies it.

Dr. Battey thought that the electrical mania of the day is but a cyclical return of the old tractors and other similar vagaries of the professional brain.

Dr. Baird remarked that it was true we as yet know but little as to the details of the action of electricity as a therapeutical agent, but did not think the subject so dark as had been suggested. We do know something. We know that, as a general rule, the constant or galvanic current is more applicable to contracted muscles than the induced or interrupted current, and that the latter is preferable for paralyzed muscles. Furthermore, that paralyzed muscles sometimes respond to the induced current after several applications of a constant current, when they would not have done so if the former had been used in the first instance. So far as spinal irritation is concerned, he distinctly believes it to be anæmia of the posterior columns of the cord. Thought electricity a valuable adjunct to the treatment of this affection. The galvanic current should be applied to the spine, the positive pole below and the negative pole above, producing an upward current for dilating the vessels of the cord. The downward current produces contraction of the vessels. Adjourned.

---

ROBERT BATTEY, M.D., REPORTER.

---

ATLANTA, GA., February 16, 1875.

Dr. V. H. Taliaferro, Vice-President, presiding.

Dr. Baird reported case of a child, aged sixteen months, accidentally shot in the os frontis, a pistol ball passing into the brain. There was, in the general symptoms, very little evidence of serious injury. Ordered quiet, and bromide potassium. On twenty-first day evacuated abscess at back of the head and let out pus copiously. Died on the twenty-fifth day. Points of interest are the absence of evidences of shock after injury, and the survival of the child for so long a period after so serious injury of the brain. No *post mortem* obtained.

Dr. Battey reported progress in his case of ovariotomy. On the fifth and sixth days the pulse ran up to 156, and the temperature was excessive, but was not noted by the thermometer. There were no symptoms, aside from the pulse and temperature, indicative of either peritonitis or septicæmia. There has been no complaint of pain in the abdomen; the abdomen is flat and not tender; bears pressure remarkably well; flatus is passed freely per anum. On the second day the bowels moved three times, rather loosely; sixth day, two rather consistent motions; seventh day two, and to-day one motion. These evacuations are fæcal and natural, though rather soft in consistency. There is nothing to be felt in the utero-rectal pouch, though there is tenderness of the uterus. She takes food and brandy well. The wound is cicatrized well; the sutures all out; the pedicle is nearly all removed. To-day (the eighth after operation) the pulse is 112, and temperature normal. On the third day she had a rather severe pleurodynia of the right side, just such as she had suffered ten days prior to operation. It was entirely relieved in twelve hours. There was no cough and the ausculatory sounds were normal, excepting a slight prolongation of the expiratory murmur, and a little, not well marked, dulness at the apex of the right lung.

Dr. Logan reported the case of a lady who had been subject to attacks of hæmorrhoids. Was consulted for painful defæcation. Made rectal examination; found thickened mucous membrane; no fissure or ulcer. Made forcible extension of the sphincter muscle and gave opiate. She was relieved temporarily, but the distress returned in a week. Pushing the investigation further, he discovered a mass of impacted fæces quite high up. This was broken down and brought away, with prompt and entire relief. Inquiry elicited the fact that the patient had been for a time in the habit of eating ground-peas, which was probably the cause of the fæcal impaction.

Dr. Woodhull reported the case of a woman of forty, with phthisical tendency. Menses had ceased for two years. A good deal of gastric irritability and daily rejection of food from the stomach for three years. He suspected a uterine trouble was at the bottom of the gastric irritability. Inquired into the state of the uterus. She had had some such trouble some years ago—not conscious of any now. He reasoned that uterine irritability might exhaust the ganglionic system of nerves and thus induce the want of tone in the stomach; he therefore put her upon one drop of wine of ipecac. every hour. Under this treatment the vomiting ceased and the appetite increased very markedly. He thought the sequence of the improvement upon the treatment very striking. He has had favorable observation of the use of the one drop dose of the wine of ipecac. in the nausea of pregnancy.

Dr. Flewellyn had observed in the treatment of dysentery that ipecac. was often salutatory, but had observed that when he had good effect from the ipecac, there was always catharsis, and suggests the inquiry whether there was not catharsis in Dr. Woodhull's case.

Dr. Woodhull replied there was not.

Dr. Battey called attention to the plan of breaking up impacted fæces in the female by bringing the mass between the firm wall of the sacrum and the two fingers passed high up into the vagina. He thinks this expedient new; it certainly is original with himself; but it is hazardous to claim anything as absolutely original at the present day. The mass by this method is easily broken up in most cases, and the disintegrated masses are pushed out at the anus into a napkin. By this method we may overcome the very great repugnance of ladies to rectal manipulations, as well as avoid the disgusting fæcal odor which clings so tenaciously to the hands of the operator.

Dr. Logan said the method was certainly new to him, and he should try it when another suitable case offered. He thought, however, in the case he had reported, the impacted mass was so firm and hard that there would be danger of injury to the recto-vaginal wall in the adoption of the expedient of Dr. Battey.

Dr. Connally related a case of fæcal impaction of long standing remedied by mechanical removal. The mass was larger than the double fist, and of the consistency of stiff putty. Adjourned.

